# Left nasal reconstruction using a concha cartilage for loss of substance post human bite: A case report

**DOI:** 10.1016/j.ijscr.2025.111052

**Published:** 2025-02-14

**Authors:** Boboe Jordan, Valimungighe Moise Muhindo, Yevide Agossou Barthelemy, Gbessi Mahugnon Emmanuel, Gbessi Dansou Gaspard, Jeannot Baanitse

**Affiliations:** a Faculty of Medicine, National University Hospital of HKM, Cotonou, Benin; b Faculty of Medicine, Université Catholique du Graben of Butembo, Democratic Republic of the Congo; c Surgery Department, Zonale Hospital of Klouékanme, Couffo, Benin; d Faculty of Medicine, La Sapientia Catholic University of Goma, Democratic Republic of the Congo; e Faculty of Clinical Medicine and Dentistry, Surgery Department, Kampala International University, Ishaka, Uganda

**Keywords:** Nasal reconstruction, Nasal labial flap, Loss of substance, Auricular cartilaginous graft, Case report

## Abstract

**Introduction and importance:**

Conchal cartilage is recommended for correction of substance loss of the nose. Transfixing nose substance loss presents an anatomical, functional, and aesthetic restoration challenge. The rate of complications associated with concha cartilage harvesting using a retroauricular approach is low.

**Case presentation:**

A 24-year-old presented with nose damage and loss of transfixing substance after being intentionally assaulted and injured (human bite during a fight). He underwent a nose wing reconstruction using a composite transplant from the auricular cartilage. Post-operative recovery was uneventful. Nonetheless, a minor disparity existed between the flap and the natural nasal wing.

**Clinical discussion:**

The three-dimensional anatomy of the nasal wing makes the aesthetic reconstruction of the transfixing loss of substance of the entire unit very difficult to achieve, especially when the reconstructed wing will be constantly confronted and compared to the contralateral wing. Concha cartilage is recommended for correction of cleft nasal deformities of trauma. Morbidities at the donor site have been reported in aesthetic rhinoplasty cases.

**Conclusion:**

The composite nose graft, which consists of a cartilaginous graft and a nasolabial flap, is still a straightforward option for reconstructing the nose wing in a remote location. Nasal reconstruction using a concha cartilage, restore the anatomically deficient structures with satisfactory aesthetic and easy to shape. The conchal cartilage is minimally invasive when harvesting, repair for large defect and conforms naturally to the curvature of the nasal wing.

## 1 Introduction

One of the most important and aesthetic organs of the face is the nose. Transfixing nose substance loss presents an anatomical, functional, and aesthetic restoration challenge. Post-traumatic rhinoplasty, is a surgical procedure that treats the sequelae of nasal trauma to improve the function and shape of the post-traumatic nose [1]. Conchal cartilage is generally favored in rhinoplasty with a satisfied aesthetic outcome [2]. The nasal rehabilitation aims at improved function and aesthetic of patients with facial deformations resulting in considerable cosmetic impairment. The aim of this report was to describe the nasal rehabilitation, in which the Retroauricular cartilaginous graft harvest from the left ear's concha [1,3]. The work has been reported in line with the SCARE criteria [4].

## 2 Clinical observation

**Fig. 1:**
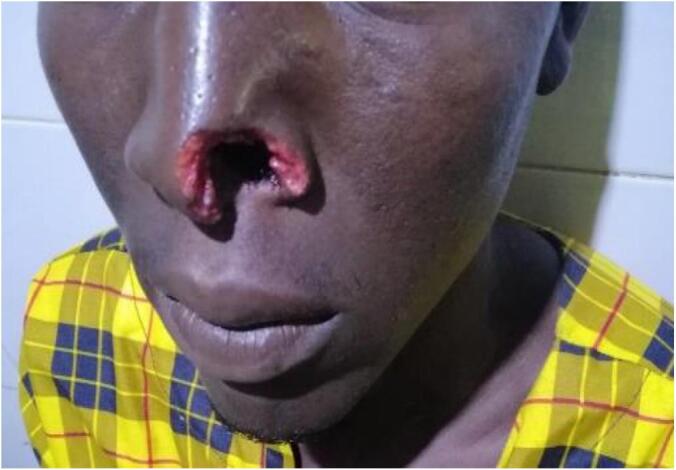
Transfixed wound of the nose's left wing after washing the wound.

A 24-year-old male patient with no specific past medical history was brought to the emergency room due to nasal trauma and substance loss by intentional assault and injury (a human bite during a fight). Two hours after the incident, he was referred to our hospital after consulting at two other health institutions. A transfixing loss of substance from the left wing of the nose, with damaged edges, measuring about 3 × 4 cm, was noted during the physical examination at admission (Fig. 1). The base of the wing was unaffected.

Additionally, we discovered abrasions on the right forearm during physical examination.

**Fig. 2:**
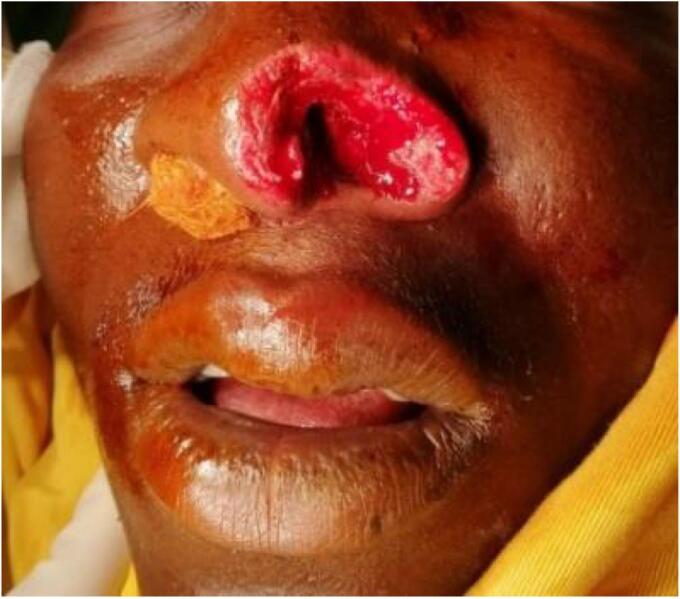
Wound after debridement.

The decision was taken to use a composite auricular cartilage transplant for nose reconstruction. He was transferred to the operating room following consent and prophylactic tetanus treatment. We debrided the lesion under local anesthesia while minimizing further tissue loss (Fig. 2).

**Fig. 3:**
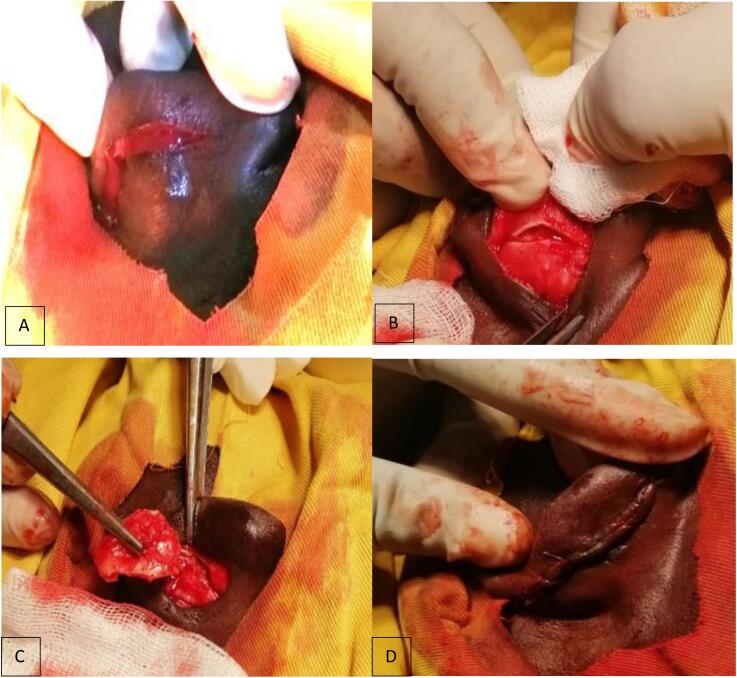
Retro-auricular cartilaginous graft harvest from the left ear's concha, where A) shows the incision through the skin of the left posterior ear. B) shows the incision through the concha cartilage. C) shows the harvest of the conchal cartilage, and D) shows the closed wound after harvesting the cartilage.

Next, a 3.5 × 1.5 cm rectangular piece of concha cartilage was harvested from the ipsilateral ear using the posterior approach (Retroauricular approach) (Fig. 3). After harvesting the concha cartilage, hemostasis was achieved using Vicryl suture 2–0 and nylon suture 2–0, using interrupted suture. Using 4/0 non-absorbable suture, the transplant was secured as a bridge to the remaining wing cartilages.

**Fig. 4:**
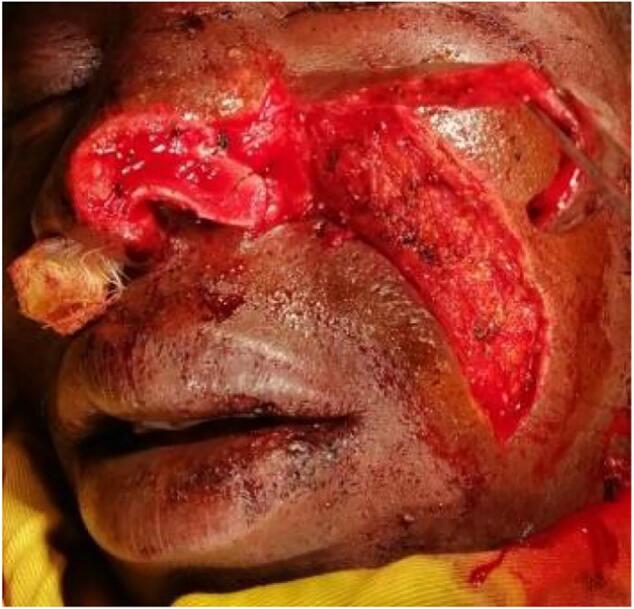
Harvesting the naso-labial flap to cover the concha cartilage.

Next, we elevated a nasolabial flap on the angular artery using a superior pedicle (Fig. 4).

**Fig. 5:**
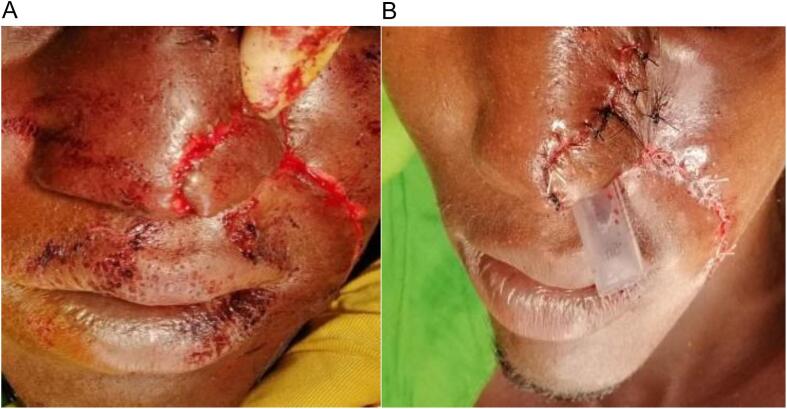
Naso-labial flap fixation with the tube inserted in the left nostril. A) shows the fixation of the naso-labial flap before suturing it. B) shows the fixed naso-labial flap with a tube inserted in left nostril to maintain the normal shape.

To restore the mucosal side, it was first degreased and then folded. Two planes (layers) of the flap were secured and sutured with 3/0 absorbable suture. 3/0 absorbable suture was used to suture the donor region in two layers. For seventy-two hours, a plastic tube was inserted into the left nostril to keep the shape of the nose (Fig. 5).

**Fig. 6:**
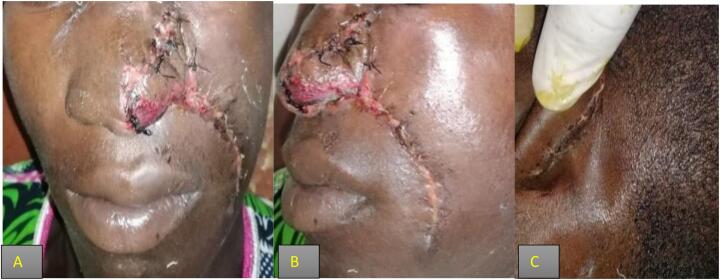
Healed naso-labial flap recipient and donor locations at D-10 post-op. A–B) show a healed naso-labial flap at Day 10 post- operative when the patient came for stitch removal. C) shows the healed wound of the harvested part in retro-auricular.

Ten days of antibiotic treatment (Ceftriaxon 2 g daily for 10 days to prevent any post -operative infection) were recommended after surgery. On the tenth day post operation, the donor area had healed satisfactorily, and the flap had successfully integrated into the nasal pyramid (Fig. 6). Nonetheless, a minor disparity existed between the flap and the natural nasal wing.

## 3 Follow up

Post-operative recovery was uneventful. The patient was discharged on post-operative day 14 after removal of stitches. The nose wound had healed properly on the 21st day after discharge, no complication occurred in the follow up.

Our study was conducted in a teaching hospital and the surgery was performed by a senior consultant general surgeon with another general surgeon as an assistant.

## 4 Discussion

Loss of substances from the nasal pyramid most often occurs following excision of skin carcinomas in subjects with a white or light phototype [3]. The three-dimensional anatomy of the nasal wing makes the aesthetic reconstruction of the transfixing loss of substance of the entire unit very difficult to achieve, especially when the reconstructed wing will be constantly confronted and compared to the contralateral wing [5]. The chondrocutaneous graft is the most judicious choice for loss of substances less than or equal to 1 cm [6,7].

The nasolabial flap, ideally reinforced by a cartilaginous frame [8], the strengthened or reinforced forehead flap or the Schmid-Meyer flap [6] and repairs in three layers: mucosal, cartilaginous and cutaneous [2,8], take the lead in losses of more important substances [3]. Classically we distinguish 2 types of nasolabial flap, either with a superior pedicle [9], as in our case, or with a lower pedicle [1,9]. Several authors describe nasolabial flap techniques such as Pers, Burget, Spear and Préaux, [8]. We used the latter technique in our case report. Because it may be finished in a single step, it is the preferred method for substance loss in the centro-facial region. Technically, raising the flap is simple and has good vascular reliability [1,5]. The nose may appear straight and well-corrected on the operating table, but may re-deviate during healing [1,11].

Complications can occur, some of a general nature, inherent to any surgical procedure, others loco-regional more specific to the reconstruction of the upper aero-digestive tracts [10]. Infection, hematoma, suture disunity, partial distal necrosis of the flap and healing disorders [10,11]. As a disadvantage, possible edema, linked to its counter-current venous vascularization may persist for several weeks. Possible retraction may be bothersome in this location where the skin is thin. Sometimes secondary degreasing may occasionally be feasible [2,9]. Concha cartilage is recommended for correction of cleft nasal deformities of trauma. Morbidities at the donor site have been reported in aesthetic rhinoplasty cases [12].

## 5 Conclusion

Nose reconstruction with a concha cartilage transplant and a nasolabial flap is still a straightforward option for reconstructing the nose's wing in a low income country. Concha cartilage is a good choice for nasal's wing reconstruction and mostly recommended.

## CRediT authorship contribution statement

BJ, YAB, GM and GB managed the patient and wrote the first draft. VM and JB helped in editing and reviewing the paper. All authors read and approved the final version to be published.

## Consent

Written informed consent was obtained from the patient for publication and any accompanying images. A copy of the written consent is available for review by the Editor-in-Chief of this journal on request.

## Ethical approval

Not applicable.

## Strength and weakness of the study

Strength of the study: clear and concise data on nasal wing reconstruction with excellent iconographies are mentioned in this case presentation.

Weakness of the study: The discussion is not detailed very well. There is no much literature search on recommendations and results from previous publications.

## Guarantor

Jeannot Baanitse.

## Research registration number

Not applicable.

## Provenance and peer review

Not commissioned, externally peer-reviewed.

## Funding

There was no external funding source for this report.

## Declaration of competing interest

The authors declare no conflict of interest.
